# Impact of arterial load on the agreement between pulse pressure analysis and esophageal Doppler

**DOI:** 10.1186/cc12785

**Published:** 2013-06-20

**Authors:** Manuel Ignacio Monge García, Manuel Gracia Romero, Anselmo Gil Cano, Andrew Rhodes, Robert Michael Grounds, Maurizio Cecconi

**Affiliations:** 1 Servicio de Cuidados Intensivos y Urgencias, Hospital SAS de Jerez, C/ Circunvalación s/n, 11407 Jerez de la Frontera, Spain; 2 Department of Intensive Care Medicine, St. George's Healthcare NHS Trust and St George's University of London, Tooting, London SW17 0QT, UK

## Abstract

**Introduction:**

The reliability of pulse pressure analysis to estimate cardiac output is known to be affected by arterial load changes. However, the contribution of each aspect of arterial load could be substantially different. In this study, we evaluated the agreement of eight non-commercial algorithms of pulse pressure analysis for estimating cardiac output (PPCO) with esophageal Doppler cardiac output (EDCO) during acute changes of arterial load. In addition, we aimed to determine the optimal arterial load parameter that could detect a clinically significant difference between PPCO and the EDCO.

**Methods:**

We included mechanically ventilated patients monitored with a prototype esophageal Doppler (CardioQ-Combi™, Deltex Medical, Chichester, UK) and an indwelling arterial catheter who received a fluid challenge or in whom the vasoactive medication was introduced or modified. Initial calibration of PPCO was made with the baseline value of EDCO. We evaluated several aspects of arterial load: total systemic vascular resistance (TSVR = mean arterial pressure [MAP]/EDCO * 80), net arterial compliance (C = EDCO-derived stroke volume/pulse pressure), and effective arterial elastance (Ea = 0.9 * systolic blood pressure/EDCO-derived stroke volume). We compared CO values with Bland-Altman analysis, four-quadrant plot and a modified polar plot (with least significant change analysis).

**Results:**

A total of 16,964-paired measurements in 53 patients were performed (median 271; interquartile range: 180-415). Agreement of all PPCO algorithms with EDCO was significantly affected by changes in arterial load, although the impact was more pronounced during changes in vasopressor therapy. When looking at different parameters of arterial load, the predictive abilities of Ea and C were superior to TSVR and MAP changes to detect a PPCO-EDCO discrepancy ≥ 10% in all PPCO algorithms. An absolute Ea change > 8.9 ± 1.7% was associated with a PPCO-EDCO discrepancy ≥ 10% in most algorithms.

**Conclusions:**

Changes in arterial load profoundly affected the agreement of PPCO and EDCO, although the contribution of each aspect of arterial load to the PPCO-EDCO discrepancies was significantly different. Changes in Ea and C mainly determined PPCO-EDCO discrepancy.

## Introduction

Cardiac output (CO) is an essential component in the hemodynamic assessment of critically ill patients and a central target for goal-directed resuscitation in high-risk surgical patients [1]. During recent years, many minimally invasive beat-to-beat methodologies have been developed to monitor CO [2]. One of the proposed approaches that has emerged is pulse pressure analysis (PPA) [3]. The basic physiological principle of these techniques is that changes in arterial pulse pressure relate directly to changes in stroke volume (SV); thus, changes in CO can be continuously estimated from analysis of the arterial waveform. However, this relationship can be affected by arterial load [4,5]; therefore, if changes in arterial load occur, the reliability of CO measurements by PPA techniques may be affected [6-10]. Although each PPA algorithm is different, they all have to tackle similar limitations. For this reason, most modern systems based on PPA use a calibration (internal or external) to determine the individual arterial load of the patient in order to convert the pressure signal into a volume-based parameter. Subsequent recalibrations are then required when significant variations in arterial load occur [6-9].

Previous studies have demonstrated that changes in arterial load can affect the reliability of pulse pressure-derived cardiac output (PPCO) measurements [6-9,11,12]; however, these studies have focused on only one aspect of the arterial load: the systemic vascular resistance. This approach ignores the pulsatile nature of the cardiovascular system and provides only a partial description of arterial load, ignoring other components such as arterial compliance or wave reflections [13].

The aim of this study was to describe the performance of PPA during arterial load changes when comparing it with an independent reference technique used as the initial calibration. We assumed that changes in arterial load would affect all PPA algorithms, although the contribution of each aspect of arterial load may be substantially different. For this purpose, we first assessed the reliability of eight non-proprietary PPA algorithms for estimating CO during the introduction or modification in dose of vasoactive medication or during intravenous volume administration. Second, we attempted to identify the optimal arterial load parameter able to predict a clinically significant discrepancy between PPCO and the esophageal Doppler cardiac output (EDCO).

## Materials and methods

### Patients

This study was performed in the 17-bed multidisciplinary intensive care unit of the Hospital de SAS Jerez from September 2011 to October 2012. The protocol was approved by the institutional research ethics committee of the Hospital del SAS de Jerez de la Frontera. Informed consent was deemed unnecessary because of the observational nature of the study and because the protocol was considered to be part of the standard care.

Inclusion criteria included any patient who was already monitored with an esophageal Doppler and indwelling arterial catheter for clinical reasons and for whom the attending physician decided to perform a fluid challenge or titrate the dosage of norepinephrine to maintain a desired level of mean arterial pressure (MAP). Patients with known severe valvular regurgitation, atrial fibrillation, or any contraindication to the use of the esophageal Doppler were excluded.

### Hemodynamic monitoring

Hemodynamic monitoring was performed by using the CardioQ-Combi™ esophageal Doppler monitor (Deltex Medical, Chichester, UK). This prototype combined a standard Doppler monitor with eight PPCO algorithms. The Doppler probe was inserted into the esophagus via the nasal or oral route and advanced until the maximal peak velocity of aortic blood flow signal was achieved. The gain setting was then adjusted to obtain the optimal outline of the aortic velocity waveform.

### Pulse pressure analysis algorithms

All patients had an arterial catheter for continuous arterial pressure monitoring. After zeroing of the system to atmospheric pressure, the arterial waveform was carefully checked by using a fast flush test in order to ensure optimal harmonics of the arterial pressure measurement system. The absence of overdamping/underdamping of the arterial pressure waveform was a prerequisite for hemodynamic measurements. The arterial pressure signal was transferred from the patient bedside monitor to the esophageal Doppler system by using a serial cable. Eight non-proprietary PPA algorithms built into the CardioQ-Combi™ software and PPCO values were continuously displayed in the monitor along with the arterial load parameters. Initial calibration of PPCO was performed by using the EDCO value before any intervention and after stabilization of heart rate and arterial pressure (less than 5% variation for a 1-minute period). A list of all PPCO algorithms analyzed in this study is shown in Table 1. A more detailed description of each algorithm can be found in the Appendix of Additional file 1. EDCO, PPCO measurements, and other hemodynamic variables were averaged and recorded every 10 seconds.

**Table 1:** Pulse pressure-derived algorithms tested

Algorithm name (source, year)	Algorithm description
Windkessel (Erlanger and Hooker [31], 1904)	k * (SBP-DBP) * HR

Windkessel with RC decay (Bourgeois *et al. *[32], 1976)	k * (MAP/T) * ln(SBP/DBP) * HR

Liljestrand-Zander (Liljestrand and Zander [33], 1928)	k * (SBP-DBP)/(SBP + DBP) * HR

Herd (Herd *et al. *[34], 1966)	k * (MAP-DBP) * HR

Pressure root-mean-square (Jonas and Tanser [35], 2002)	$\text{k}*\sqrt{\int_{\text{T}}{\left(\text{ABP}\left(\text{t}\right)-\text{MAP}\right)}^{2}\text{dt}}*\text{HR}$

Systolic area (Jones *et al. *[36], 1959; Verdouw *et al. *[37], 1975)	$\text{k}*\int_{\text{sys}}\text{ABP}\left(\text{dt}\right)*\text{HR}$

Systolic area with correction (Warner *et al. *[38], 1953; Kouchoukos *et al. *[39], 1970)	$\text{k}*\int_{\text{sys}}\text{ABP(dt)}*\left(1+\frac{\text{Tsys}}{\text{Tdia}}\right)*\text{HR}$

Corrected impedance (Wesseling *et al. *[40], 1983; Rauch *et al. *[41], 2002)	$\text{k}*(163+\text{HR}-0.48*\text{MAP)*}\int_{\text{sys}}\text{ABP}(\text{dt})*\text{HR}$

ABP, arterial blood pressure; DBP, diastolic blood pressure; HR, heart rate; k, calibration factor obtained from esophageal Doppler cardiac output; MAP, mean arterial pressure; SBP, systolic blood pressure; T, duration of cardiac cycle (T = √HR/60); T_dia_, duration of diastole (T_dia _= T − T_sys_); T_sys_, duration of systole (estimated as a 30% of cardiac cycle time).

### Arterial load assessment

Considering the pulsatile nature of arterial blood, we evaluated different aspects of arterial load [14]. The steady component was computed by using the total systemic vascular resistance (TSVR), defined as MAP divided by the product of EDCO times 80. The pulsatile component was calculated by using the net arterial compliance (C), defined as EDCO-derived SV divided by arterial pulse pressure. Although this approach, by definition, overestimates the real compliance and violates the fundamental concept of the Windkessel model (since it assumes that the total SV is buffered in the large elastic arteries during systole without any peripheral outflow), it has been shown to be a useful index for the estimation and detection of relative changes in arterial compliance [15-17]. We also calculated the effective arterial elastance (Ea), an integrative parameter that incorporates both the steady and pulsatile components of arterial load, including resistance, compliance, characteristic impedance, and cardiac cycle time intervals [14,18,19]. Effective arterial elastance was computed as Ea = 0.9 * systolic arterial pressure (SAP)/EDCO-derived SV, where the 90% of SAP was used as a surrogate of left ventricular end-systolic pressure [18,20].

### Study protocol

Therapeutic interventions were made only according to the decisions of attending physicians. Clinical care was guided by local protocols and the information obtained via EDCO. Subgroups were defined according to patients receiving fluid administration or introduction/modification in vasoactive dose. Fluid administration consisted of 500-mL infusions of crystalloid for a 25- to 30-minute period. Vasopressor changes consisted of stepwise increases or decreases in norepinephrine in 2- to 4-μg/minute increments every 2 to 3 minutes to maintain a desired MAP level. Supportive therapies and ventilatory settings remained constant and vasopressors (if any) unchanged during fluid administration. All patients were under controlled mechanical ventilation.

### Statistical analysis

Normal distribution of data was tested by using the D'Agostino-Pearson test. The results are expressed as mean ± standard deviation (SD) or as median and interquartile range (IQR), as appropriate. Bland-Altman analysis for repeated measurements was used to assess the agreement between EDCO and each PPCO algorithm [21]. Bias was defined as the mean difference between EDCO and PPCO measurements. Limits of agreement (LOAs) were calculated as mean bias ± 1.96 SD. Percentage error (PE) was calculated for comparison of CO as PE (%) = LOA/[(mean EDCO + mean PPCO)/2]. Coefficient of variation (CV) of EDCO measurements was determined during a 1-minute period at baseline during stable hemodynamic conditions in all patients. As suggested by Cecconi and colleagues [22], the precision of the reference technique was calculated as 2 times CV, and the least significant change (LSC), the minimum change between successive measurements that can be considered a real change and not due to random error, was calculated as LSC = precision × √2.

The ability to track EDCO changes by each PPCO algorithm was tested by using a concordance analysis [23]. Concordance was defined as the percentage of data in which the direction of change agreed in four-quadrant plots or as the percentage of data within ± 30° radial limits of agreement (RLOA = *θ *± 1.96 SD) in polar plots, according to the method proposed by Critchley and colleagues [24]. This method accounts not only the direction but also the magnitude of CO change to assess trending ability. Acceptable concordance was assumed when the concordance rate was more than 90% in four-quadrant plots or when the mean angular bias (that is, the mean angle of all radial vectors from the polar axis) and RLOA were less than ± 5° and less than 30%, respectively. To exclude random measurement error, a central exclusion zone was selected for analysis. We tested a new approach by implementing the concept of LSC in each polar plot, by suggesting that the exclusion zone size should be calculated using the combined LSC for EDCO and PPCO. Assuming an LSC_PPCO _equal to LSC_EDCO_, the combined LSC was calculated as √[2 × (LSC_EDCO_)^2^] for selecting the exclusion zone size.

The relationship between changes in arterial load parameters and discrepancy between EDCO and each PPCO algorithm [EDCO-PCCO discrepancy (%) = (PPCO − EDCO)/EDCO] was assessed by a regression analysis. A discrepancy of at least 10% between techniques after initial calibration was considered clinically significant. For each PPCO algorithm, a receiver operating characteristic (ROC) curve was also constructed for testing the ability of absolute percentage changes on each arterial load parameter to predict an absolute PPCO-EDCO discrepancy of at least 10%. Areas under the ROC curves were compared by using the method described by DeLong and colleagues [25]. Differences between groups were compared by the Mann-Whitney *U *test or an independent Student *t *test for independent samples, as appropriate. A *P *value of less than 0.05 was considered statistically significant. All statistical analyses were two-tailed and performed using MedCalc for Windows version 12.3.0 (MedCalc Software bvba, Mariakerke, Belgium).

## Results

Fifty-three consecutive patients were included. Patient characteristics are summarized in Table 2. Thirty-five patients (66%) had sepsis as defined by standard criteria [26]. In total, 16,964 paired measurements were performed (median 271, IQR 180 to 415). Forty-five fluid challenges were performed in 38 patients, and 19 of the fluid challenges (42%) induced an EDCO increase of at least 10%. The main reason for volume administration was oliguria (95%). Forty-seven changes in dose or introduction of vasopressors were performed in 36 patients. Baseline CO was 6.3 ± 2.9 L/minute, heart rate was 92 ± 21 beats per minute, and MAP was 70 ± 12 mm Hg.

**Table 2:** Characteristics and demographic data of study population

Age, years	60.1 ± 15.2
Gender, males/females	37/16

Weight, kg	80 (70 to 90)

Height, cm	169.6 ± 8.3

ICU mortality rate, number (%)	14 (26.4)

Vasoactive agents at inclusion time, number (dose, µg/kg per minute)	

Norepinephrine	38 (0.16; 0.09 to 0.28)

Dobutamine	5 (8.33; 7.18 to 9.59)

Analgesia and sedative drugs	

Fentanyl, n (dose, µg/kg per hour)	40 (1.44; 1.07 to 2)

Remifentanil, n (dose, µg/kg per minute)	8 (0.09; 0.08 to 0.12)

Morphine, n (dose, mg/hour)	2 (2 to10)

Midazolam, n (dose, mg/kg per hour)	42; 0.09 ± 0.03

Propofol, n (dose, mg/kg per minute)	6 (0.92; 0.59 to 1.54)

Ventilator settings	

Tidal volume, mL/kg predicted body weight	7.9 (7.2 to 8.8)

Respiratory rate, breaths per minute	18.6 ± 1.6

Total PEEP, cm H_2_O	6 (6 to 8)

FiO_2_, %	66.5 ± 17.8

SaO_2_, %	99 (97 to 99)

Arterial catheter site, radial/femoral	46/7

Acute respiratory distress syndrome, n	6

Reason for admission, n	

Sepsis/Septic shock	

Abdominal	22

Pulmonary	10

Urological	2

Neurological	1

Cardiogenic shock	5

Hemorrhagic shock	6

Mesenteric thrombosis	1

Acute cerebrovascular disease	4

Polytrauma	3

Values are expressed as mean ± standard deviation, median (25th to 75th percentile), or absolute numbers, as appropriate. FiO_2_, inspired oxygen fraction; ICU, intensive care unit; PEEP, positive end-expiratory pressure; SaO_2_, arterial oxygen saturation.

Overall, the median EDCO value was 6.3 L/minute (IQR 4.9 to 7.2 L/minute), and absolute percentage change throughout all interventions was 4.9% (IQR 2.1% to 9.8%) with a range of change from −46.9% to 57.7%. During the introduction or modification in vasoactive dose, the absolute percentage change for EDCO was 4.5% (IQR 1.8% to 9.7%) with a range of change from −46.9% to 37.7%. After volume administration, EDCO increased 9.5% (IQR 3.8% to 16.3%).

The mean CV for EDCO measurements was 2.4% ± 1.3%. The precision and LSC for EDCO were 4.7% and 6.7%, respectively. According to the combined LSC for both techniques, we selected a 9.5% value for central exclusion zone in the concordance analysis.

### Reliability of pulse pressure analysis during arterial load changes

**Figure 1:**
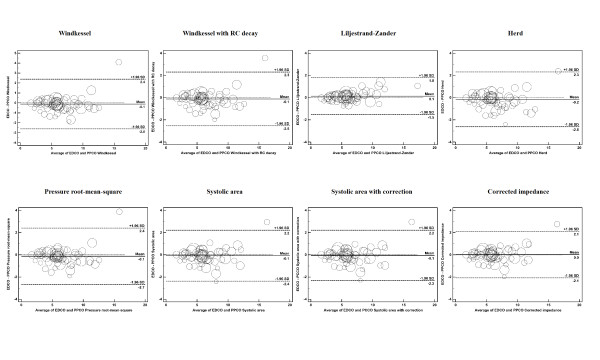
**Bland-Altman plots for the absolute values of pulse pressure-derived cardiac output (PPCO) versus esophageal Doppler cardiac output (EDCO)**. Agreement between PPCO and EDCO measurements according to Bland-Altman analysis is shown. Only one marker for subject is represented in the graph. The marker size is relative to the number of observations per subject. Solid lines represent bias (mean difference between EDCO and PPCO measurements). Dashed lines are the upper and lower limits of agreement: bias ± 1.96 standard deviation (SD).

**Figure 2:**
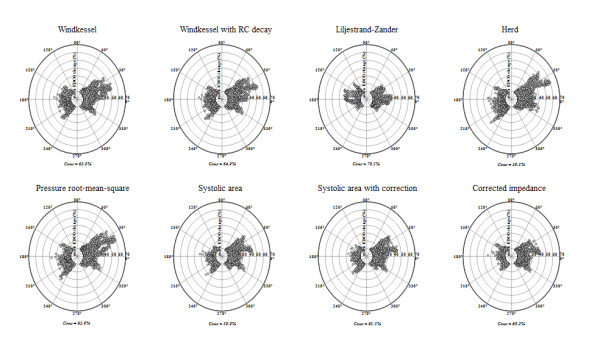
**Trending ability of pulse pressure-derived cardiac output (PPCO) versus esophageal Doppler cardiac output (EDCO) based on polar plot analysis**. Concordance analysis based on polar plots for evaluating trending ability of PPCO versus EDCO (agreement in direction and magnitude of change). Exclusion zone was 9.5% (central white circle). The magnitude of polar vector (the distance from the center of polar plot) represents the mean change in cardiac output. The angle of polar vector with the horizontal axis (θ) is the agreement between both methods. Good trending capability was assumed when most of the data lie within ± 30° radial limits of agreement. Conc, concordance rate.

A detailed description of the agreement analysis between EDCO and all PPCO algorithms is shown in Table 3. Bland-Altman plots for differences between absolute values of EDCO and PPCO are presented in Figure 1. Subgroup analyses are reported in Figures 1 and 2 of Additional file 1. The best agreement was for the Liljestrand-Zander algorithm in all conditions (bias ± LOA: 0.1 ± 1.7 L/minute, PE 27.2%), whereas the Pressure root-mean-square Algorithm performed the worst (bias ± LOA: −0.1 ± 2.5 L/minute, PE 40.8%). The mean bias, LOA, and PE were significantly higher in the vasopressor group (*P *<0.01 versus patients receiving fluid administration). There were no significant differences between septic and non-septic patients in bias (−0.03 versus −0.13 L/minute, *P *= 0.06) and PE (37.76% versus 34.08%, *P *= 0.14). However, LOA was wider in the septic group (2.36 versus 1.87 L/minute, *P *<0.01; Table 1 of Additional file 1).

**Table 3:** Agreement and concordance analyses between esophageal Doppler and pulse pressure-derived algorithms for estimation of cardiac output and tracking EDCO changes

PPCO algorithm	Mean CO, L/minute	Bias ± LOA, L/minute	PE, percentage	Four-quadrant plot	Polar plot	
				
				Concordance, percentage	Concordance, percentage	Mean θ ± RLOA, degrees
Windkessel	6.40	−0.11 ± 2.50	40.2	76.8	63.8	12.5 ± 52.7

Fluid administration	6.30	−0.01 ± 1.67	27.0	93.4	74.9	6.5 ± 47.5

Vasopressor change	6.59	−0.29 ± 3.15	49.8	51.8	44.7	22.9 ± 55.1

Windkessel with RC decay	6.41	−0.12 ± 2.42	38.9	77.2	64.9	12.0 ± 52.5

Fluid administration	6.31	−0.02 ± 1.68	27.2	93.6	76.8	6.5 ± 46.7

Vasopressor change	6.60	−0.31 ± 2.99	47.3	51.0	43.4	21.9 ± 57.5

Liljestrand-Zander	6.16	0.13 ± 1.66	27.2	92.3	70.5	−5.6 ± 48.8

Fluid administration	6.07	0.22 ± 1.55	25.5	91.4	64.0	−3.9 ± 53.4

Vasopressor change	6.32	−0.03 ± 1.70	27.5	94.1	84.4	−9.2 ± 35.9

Herd	6.45	−0.16 ± 2.47	39.6	76.1	59.5	13.1 ± 55.4

Fluid administration	6.35	−0.06 ± 1.89	30.6	91.7	64.5	9.9 ± 49.1

Vasopressor change	6.63	−0.34 ± 2.91	46.0	48.2	39.2	19.4 ± 64.4

Pressure root-mean-square	6.42	−0.13 ± 2.54	40.8	75.6	63.9	13.6 ± 52.6

Fluid administration	6.31	−0.02 ± 1.69	27.4	92.1	77.7	7.2 ± 44.8

Vasopressor change	6.63	−0.34 ± 3.21	50.7	45.7	40.1	24.5 ± 57.9

Systolic area	6.37	−0.08 ± 2.29	36.9	66.6	59.9	6.1 ± 62.7

Fluid administration	6.19	0.10 ± 1.49	24.4	88.5	75.1	−3.9 ± 50.7

Vasopressor change	6.69	−0.39 ± 2.88	45.3	36.6	35.3	22.2 ± 67.1

Systolic area with correction	6.34	−0.05 ± 2.27	36.7	66.3	61.5	4.9 ± 62.5

Fluid administration	6.17	0.12 ± 1.55	25.3	86.1	74.7	−3.7 ± 51.2

Vasopressor change	6.66	−0.37 ± 2.78	43.8	38.4	35.8	19.2 ± 69.1

Corrected impedance	6.27	0.02 ± 2.10	34.1	65.4	60.2	−1.8 ± 60.3

Fluid administration	6.10	0.19 ± 1.50	24.7	80.3	70.4	−8.5 ± 50.9

Vasopressor change	6.60	−0.31 ± 2.48	39.2	44.9	41.9	10.2 ± 68.3

Concordance refers to the percentage of agreement between changes between esophageal Doppler cardiac output (EDCO) and pulse-pressure derived cardiac output (PPCO) measurements (exclusion zone = 9.5%, obtained from the combined least significant change for EDCO and PPCO). CO, cardiac output; LOA, limits of agreement; mean θ, mean angle of all radial vectors from the polar axis; PE, percentage of error = 2 standard deviations/mean of pulse pressure-derived and esophageal Doppler cardiac output measurements; RLOA, radial limits of agreement.

The concordance between changes of PPCO and EDCO during fluid administration was good for all algorithms according to the four-quadrant plots (90% ± 5%), except for those based on the assessment of systolic area (Table 3). The concordance between changes of PPCO and EDCO during introduction or changes in dosage of vasopressors was poor for all algorithms (51% ± 18%), except for the Liljestrand-Zander (95.5%). When both the magnitude and the direction of changes were analyzed, the concordance decreased to 63.5% ± 4% with RLOA of 55.9 ± 5.2° (Table 3 and Figure 2).

### Relationship between pulse pressure-derived cardiac output and arterial load changes

**Figure 3:**
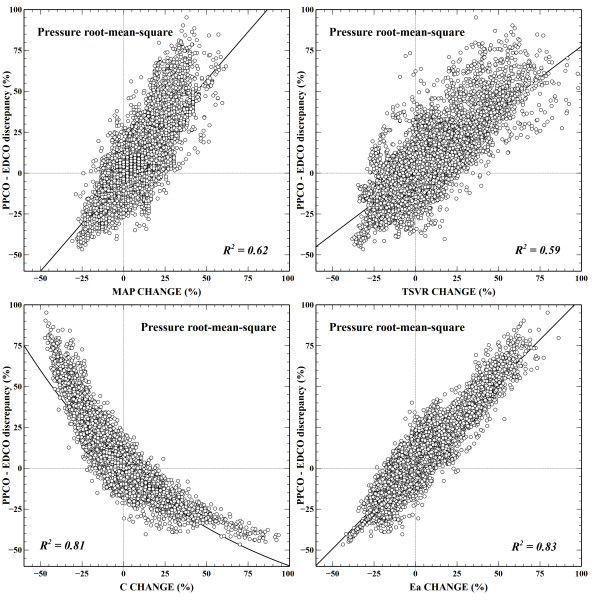
**Example of influence of mean arterial pressure, total systemic vascular resistance, net arterial compliance, and effective arterial elastance changes on discrepancies between pulse pressure-derived cardiac output (PPCO) and esophageal Doppler cardiac output (EDCO)**. PPCO-EDCO discrepancy (expressed as a percentage) is calculated by the formula (PPCO − EDCO)/EDCO. C, net arterial compliance; Ea, effective arterial elastance; MAP, mean arterial pressure; TSVR, total systemic vascular resistance.

Changes in arterial load parameters throughout different interventions are described in Table 4. Changes were more pronounced during introduction or modifications in vasopressor dosage. The contribution of Ea and C changes to PPCO-EDCO differences was superior to TSVR and MAP changes in all PPA algorithms (Figures 3 to 6 of Additional file 1). The Liljestrand-Zander algorithm was the less influenced by arterial load changes. An example of the influence of arterial load variations on discrepancies between one of the PPCO algorithms and EDCO measurements is shown in Figure 3.

**Table 4:** Changes on arterial load parameters and mean arterial pressure throughout different interventions

	Absolute percentage change	Range of percentage change
**Mean arterial pressure**		

Fluid administration	4.29 (1.7 to 8.3)	−18.3 to 44.8

Vasopressor change	11.6 (3.3 to 16.5)^a^	−30.9 to 61.6

**Total systemic vascular resistance**		

Fluid administration	5.5 (2.5 to 9.9)	−31.1 to 59.7

Vasopressor change	12.1 (5.2 to 24.5)^a^	−38.1 to 132.1

**Net arterial compliance**		

Fluid administration	6.1 (2.7 to 11.3)	−46.5 to 57.6

Vasopressor change	11.4 (5.1 to 21.4)^a^	−47.4 to 102.2

**Effective arterial elastance**		

Fluid administration	4.9 (2.4 to 9.4)	−34.4 to 79.6

Vasopressor change	10.3 (4.1 to 20.8)^a^	−43.7 to 86.2

Data are expressed as median (25th to 75th interquartile range) or as range. ^a^*P *<0.0001 versus fluid administration.

The ability of absolute percentage changes of each arterial load parameter to detect an absolute PPCO-EDCO discrepancy of at least 10% is detailed in Table 2 of Additional file 1. ROC curves for arterial load parameters on individual PPCO algorithms are shown in Figure 7 of Additional file 1. Areas under ROC curves for each arterial load parameter were significantly different in all algorithms. Overall, the performance of Ea and C changes to predict a PPCO-EDCO discrepancy of at least 10% was superior to TSVR and MAP changes in all algorithms. The performance of Ea changes was better in algorithms based on assessment of systolic area, whereas the ability of C changes to detect a significant PPCO-EDCO difference was superior in Windkessel, Herd, and Pressure root-mean-square Algorithms. However, the overall performance of Ea changes was superior to other arterial load parameters (Table 5). As long as the absolute percentage Ea change was less than 7% to 11%, the EDCO-PPCO difference was less than 10%.

**Table 5:** Pooled predictive performance of absolute percentage changes in arterial load parameters on all PPCO algorithms to detect an absolute PPCO-EDCO discrepancy of at least 10%

	Pooled AUC (95% CI)	Pooled sensitivity (95% CI)	Pooled specificity (95% CI)	Pooled optimal cutoff	Pooled Youden index
ΔMAP	0.77 (0.75-0.77)	65.2% (63.9%-66.4%)	76.8% (76.0%-77.6%)	7.4% ± 1.1%	0.42 ± 0.06

ΔTSVR	0.79 (0.78-0.79)	63.1% (61.9%-64.1%)	82.1% (81.3%-82.8%)	10.6% ± 1.3%	0.45 ± 0.14

ΔC	0.83 (0.83-0.84)	72.6% (71.4%-73.7%)	82.5% (81.8%-83.2%)	10.3% ± 0.9%	0.55 ± 0.12

ΔEa	0.86 (0.85-0.86)	75.1% (73.9%-76.2%)	83.3% (82.6%-83.9%)	8.9% ± 0.6%	0.58 ± 0.14

AUC, area under the receiver operating characteristic curve; C, net arterial compliance; CI, confidence interval; Ea, effective arterial elastance; EDCO, esophageal Doppler cardiac output; MAP, mean arterial pressure; PPCO, pulse pressure-derived cardiac output; TSVR, total systemic vascular resistance.

## Discussion

In this study, we observed that PPCO agreement with EDCO was greatly affected by changes in arterial load, although the contribution of each aspect of arterial load to the observed discrepancies was significantly different. Changes in Ea and C mainly determined PPCO-EDCO discrepancies, whereas the impact of TSVR and MAP changes was lower. In practice, changes in traditional indices such as TSVR or MAP fail to identify when EDCO and PPCO diverge. On the other hand, the performance of new indices such as Ea and C is significantly better.

To our knowledge, this is the first study to demonstrate that (a) arterial load changes during changes in vasopressor therapy and fluid administration significantly affect the agreement between EDCO and PPCO; (b) during fluid loading, arterial load changes are less pronounced than during changes in vasopressor therapy; (c) during fluid administration, EDCO and PPCO algorithms not based on systolic pressure analysis are interchangeable; (d) during vasopressor therapy, the agreement between EDCO and PPCO is severely affected; (e) precision of PPCO algorithms is more affected in patients with sepsis; and (f) the LSC can implement the power of the polar plot concordance analysis.

We believe that from our study we can draw some important information that helps us to understand how EDCO and several PPCO algorithms perform when used at the same time. We have observed that changes in the arterial load (mainly, effective arterial elastance and net arterial compliance) were major determinants in discrepancies between EDCO and PPCO. This is the first study to demonstrate that, during a fluid challenge, EDCO and PPCO perform similarly as long as the arterial load does not change. This finding brings strength to the current practice of using less invasive devices to monitor changes in CO during fluid administration [1,27]. However, when the magnitude of changes was examined, the concordance between PPCO and EDCO decreased. In practice, although trending ability seems similar, one should note that different techniques might measure different magnitudes of changes in CO.

After changes in vasopressors therapy, the agreement between EDCO and PPCO was significantly affected. This finding can generate important research hypotheses regarding the need of external calibrations for less invasive devices. Our study suggests that arterial load changes may provide information about when to recalibrate a device. This could represent an important finding that needs to be verified with protocols aiming at recalibrating when a change in arterial load has been observed.

Interestingly, our results suggest that the precision of PPA is more affected in patients with sepsis. This finding is consistent with recently demonstrated central-to-peripheral vascular tone decoupling in experimental endotoxic shock [28]. Decoupling of aortic and radial arterial pressure may be responsible for degrading the reliability of PPA in estimating CO when assessed at peripheral sites in this clinical condition.

In our study, we arbitrarily used the EDCO as our reference technique and we performed an initial calibration of PPCO based on EDCO values. The poor agreement after significant changes in arterial load could be explained as follows: (a) PPCO is significantly affected by these changes and is not reliable, (b) EDCO is significantly affected by these changes and is not reliable, or (c) PPCO and EDCO are both partially affected by these changes. We believe that the third conclusion represents the most likely explanation, but the lack of an additional external calibration prevents us from speculating any further about which technique would be affected more significantly. We argue that our findings support the recommendation of using an external calibration when significant changes in arterial load are suspected [29]. It would be important to test this hypothesis by performing a similar experiment while using an external calibration (that is, pulmonary thermodilution or transpulmonary dilution techniques) when assessing the agreement of different CO monitors after changes in vasopressor therapy.

Another important finding of this study is that the LSC calculation as suggested by Cecconi and colleagues [22,30] can be used to implement the concordance analysis when looking at changes in CO and agreement between different techniques. We suggest that this approach be used in further studies comparing different CO monitors.

There are several limitations to our study which need to be addressed. First, our comparison did not include any commercial PPCO algorithms. Nevertheless, although commercially available algorithms have significant differences, they are based on similar assumptions and thus, in theory, are susceptible to similar shortcomings [3,5]. Second, we used the EDCO as our reference technique. This is a well-validated technique, although we believe that an external calibration would clear some of the doubts from our article. However, during rapid changes (such as a fluid challenge), there is no external calibration that would allow changes to be recorded. Last, we used a processed arterial pressure signal from the bedside patient monitor and this could have significantly affected the quality of the arterial waveform and the reliability of PPCO measurements. Moreover, most of our PPCOs were connected to a radial arterial line. Although this is the most common practice, we cannot exclude the possibility that the performance may have been different at other sites.

## Conclusions

Changes in arterial load during changes in vasopressor dosage or fluid administration profoundly affect the agreement between EDCO and PPCO. Arterial load changes are less pronounced during fluid loading and this may explain the better agreement between EDCO and PPCO after a fluid challenge. The impact in EDCO-PPCO discrepancy is not equal for all arterial load parameters, the Ea and the C being the most influential variables in all studied algorithms. As long as the absolute Ea change is less than 7% to 11%, the EDCO and PPCO seem interchangeable. The LSC can be used to identify the best cutoff in a concordance analysis.

## Key messages

• The agreement between all PPCO algorithms and EDCO was profoundly affected by arterial load changes induced by changes in vasopressor therapy and fluid administration.

• The precision of PPCO algorithms was more affected in patients with sepsis.

• The contribution of net arterial compliance and effective arterial elastance to the EDCO-PPCO discrepancies was superior to TSVR and MAP changes in all PPCO algorithms.

• An absolute Ea change of more than 7% to 11% was associated with a PPCO-EDCO discrepancy of at least 10%.

## Abbreviations

C: net arterial compliance; CO: cardiac output; CV: coefficient of variation; Ea: effective arterial elastance; EDCO: esophageal Doppler-derived cardiac output; IQR: interquartile range; LOA: limits of agreement; LSC: least significant change; MAP: mean arterial pressure; PE: percentage of error; PPA: pulse pressure analysis; PPCO: pulse pressure-derived cardiac output; RLOA: radial limits of agreement; ROC: receiver operating characteristic; SAP: systolic arterial pressure; SD: standard deviation; SV: stroke volume; TSVR: total systemic vascular resistance.

## Competing interests

MIMG is a consultant for Edwards Lifesciences (Irvine, CA, USA) and has received travel expenses from Deltex Medical. AGC has received honoraria from Edwards Lifesciences. AR has received honoraria from and serves on an advisory board for LiDCO (London, UK) and has received honoraria from Covidien (Dublin, Ireland), Edwards Lifesciences, and Cheetah Medical (Vancouver, WA, USA). MC in the last 5 years has received honoraria or travel expenses or both from Edwards Lifesciences, LiDCO, Cheetah Medical, Bmeye (Amsterdam, The Netherlands), Masimo (Neuchatel, Switzerland), and Deltex Medical. MGR and RMG declare that they have no competing interests. Except for the esophageal Doppler monitor, all support was provided solely from institutional or departmental sources or both.

## Authors' contributions

MIMG conceived and designed the study, participated in the recruitment of patients, performed the statistical analysis, interpreted the data, and wrote the manuscript. MGR participated in the study design, patient recruitment, and data collection; provided technical support; and contributed to the critical review of the manuscript. AGC participated in the study conception and design, interpreted data, and helped draft the manuscript. AR and RMG made substantial contributions to the analysis and interpretation of data, were involved in drafting the manuscript, and contributed to its critical review. MC contributed in the study design, helped in the statistical analysis, interpreted data, and wrote and reviewed the manuscript. All authors read and approved the final manuscript.

## Supplementary Material

Additional file 1 **Additional Tables, Figures and Appendix**. Subgroup analyses, impact of individual arterial load parameters and PPA algorithms description. Click here for file
