# Supplementary material for: Impact of arterial load on the agreement between pulse pressure analysis and esophageal Doppler
Source: Crit Care. 2013 Jun 20;17(3):R113. doi: 10.1186/cc12785 (PMC4056096; doi:10.1186/cc12785)
Supplement: Additional file 1 — Additional Tables, Figures and Appendix. Subgroup analyses, impact of individual arterial load parameters and PPA algorithms description. [file cc12785-S1.DOCX]

**Impact of arterial load on the agreement between pulse pressure analysis and esophageal Doppler**

Manuel Ignacio Monge García (1,2), Manuel Gracia Romero (1), Anselmo Gil Cano (1), Andrew Rhodes (2), Robert Michael Grounds (2), Maurizio Cecconi (2)

1. Servicio de Cuidados Intensivos y Urgencias, Hospital SAS de Jerez, Jerez de la Frontera, Spain.
2. Department of Intensive Care Medicine, St. George’s Healthcare NHS Trust and St George’s University of London, London, United Kingdom.

**ELECTRONIC SUPPLEMENTARY MATERIAL**

| **Table 1. Comparison of agreement between esophageal Doppler and pulse pressure-derived algorithms for estimation of cardiac output in septic and non-septic patients.** | | | |
| --- | --- | --- | --- |
| **PPCO algorithm** | **Mean CO**  **(L/min)** | **Bias ± LOA (L/min)** | **PE (%)** |
| **Windkessel** |  |  |  |
| Septic | 6.69 | -0.04 ± 2.70 | 41.4 |
| Non-septic | 5.80 | -0.26 ± 2.04 | 36.8 |
| **Windkessel with RC decay** |  |  |  |
| Septic | 6.71 | -0.07 ± 2.60 | 39.8 |
| Non-septic | 5.78 | -0.24 ± 2.03 | 36.6 |
| **Liljestrand-Zander** |  |  |  |
| Septic | 6.48 | 0.16 ± 1.84 | 28.6 |
| Non-septic | 5.47 | 0.07 ± 1.21 | 22.4 |
| **Herd** |  |  |  |
| Septic | 6.80 | -0.16 ± 2.66 | 40.3 |
| Non-septic | 5.70 | -0.16 ± 2.06 | 37.1 |
| **Pressure root-mean-square** |  |  |  |
| Septic | 6.73 | -0.09 ± 2.76 | 42.1 |
| Non-septic | 5.76 | -0.22 ± 2.06 | 37.3 |
| **Systolic area** |  |  |  |
| Septic | 6.69 | -0.05 ± 2.46 | 37.7 |
| Non-septic | 5.67 | -0.13 ± 1.93 | 35.2 |
| **Systolic area with correction** |  |  |  |
| Septic | 6.67 | -0.03 ± 2.43 | 37.3 |
| Non-septic | 5.64 | -0.10 ± 1.93 | 35.3 |
| **Corrected impedance** |  |  |  |
| Septic | 6.60 | 0.04 ± 2.26 | 34.9 |
| Non-septic | 5.58 | -0.04 ± 1.74 | 32.0 |
| CO: cardiac output; LOA: limits of agreement; PPCO: pulse pressure-derived cardiac output. PE: percentage of error = 2SD/mean of pulse pressure-derived and esophageal Doppler cardiac output measurements. | | | |

| **Table 2. Predictive performance of absolute changes in arterial load parameters for all PPCO algorithms to detect an absolute percentage PPCO-EDCO discrepancy ≥ 10%. Values are AUC (95% IC) for each arterial load parameter on all PPCO algorithms.** | | | | |
| --- | --- | --- | --- | --- |
| **PPCO algorithm** | **ΔMAP** | **ΔTSVR** | **ΔC** | **ΔEa** |
| **Windkessel** | 0.793 (0.787 – 0.799) | 0.759 (0.752 – 0.765) | 0.910 (0.905 – 0.914) | 0.859 (0.854 – 0.864) |
| **Windkessel with RC decay** | 0.801 (0.795 – 0.807) | 0.768 (0.761 – 0.774) | 0.908 (0.903 – 0.912) | 0.859 (0.853 – 0.864) |
| **Liljestrand-Zander** | 0.669 (0.662 – 0.676) | 0.659 (0.652 – 0.666) | 0.707 (0.700 – 0.714) | 0.717 (0.710 – 0.724) |
| **Herd** | 0.763 (0.757 – 0.770) | 0.728 (0.773 – 0.785) | 0.833 (0.827 – 0.838) | 0.779 (0.773 – 0.785) |
| **Pressure root-mean-square** | 0.802 (0.796 – 0.808) | 0.844 (0.751 – 0.764) | 0.878 (0.873 – 0.883) | 0.844 (0.839 – 0.850) |
| **Systolic area** | 0.786 (0.780 – 0.792) | 0.875 (0.870 – 0.880) | 0.825 (0.819 – 0.831) | 0.925 (0.921 – 0.929) |
| **Systolic area with correction** | 0.778 (0.772 – 0.784) | 0.874 (0.869 – 0.879) | 0.815 (0.809 – 0.820) | 0.941 (0.937 – 0.944) |
| **Corrected impedance** | 0.747 (0.854 – 0.865) | 0.860 (0.854 – 0.865) | 0.803 (0.797 – 0.809) | 0.927 (0.923 – 0.931) |
| AUC: area under the ROC curve; C: net arterial compliance; Ea: effective arterial elastance; MAP: mean arterial pressure; EDCO: esophageal Doppler cardiac output; PPCO: pulse pressure-derived cardiac output; TSVR: total systemic vascular resistance.  Comparison of AUC for each arterial load parameter was significantly different in all PPCO algorithms according to De Long et al. method. | | | | |

| **Figure 1. Bland-Altman plots for EDCO vs. PPCO during dosage changes or introduction of vasopressors.** | | | |
| --- | --- | --- | --- |
| **Windkessel** | **Windkessel with RC decay** | **Liljestrand-Zander** | **Herd** |
|  |  |  |  |
|  |  |  |  |
| **Pressure root-mean-square** | **Systolic area** | **Systolic area with correction** | **Corrected impedance** |
|  |  |  |  |
| Agreement between PPCO algorithm and pooled CO according to Bland-Altman analysis. Only one marker for subject was represented in the graph. The marker size is relative to the number of observation per subject. Solid lines represent bias (mean difference between EDCO and pulse pressure-derived cardiac output measurements). Dashed lines are the upper and lower limit of agreement (bias ± 1.96 standard deviation). | | | |

| **Figure 2. Bland-Altman plots for EDCO vs. PPCO during fluid administration.** | | | |
| --- | --- | --- | --- |
| **Windkessel** | **Windkessel with RC decay** | **Liljestrand-Zander** | **Herd** |
|  |  |  |  |
|  |  |  |  |
| **Pressure root-mean-square** | **Systolic area** | **Systolic area with correction** | **Corrected impedance** |
|  |  |  |  |
| Agreement between PPCO algorithm and pooled CO according to Bland-Altman analysis. Only one marker for subject was represented in the graph. The marker size is relative to the number of observation per subject. Solid lines represent bias (mean difference between EDCO and pulse pressure-derived cardiac output measurements). Dashed lines are the upper and lower limit of agreement (bias ± 1.96 standard deviation). | | | |

| **Figure 3. Influence of mean arterial pressure changes on discrepancies between PPCO and EDCO.** |
| --- |
| **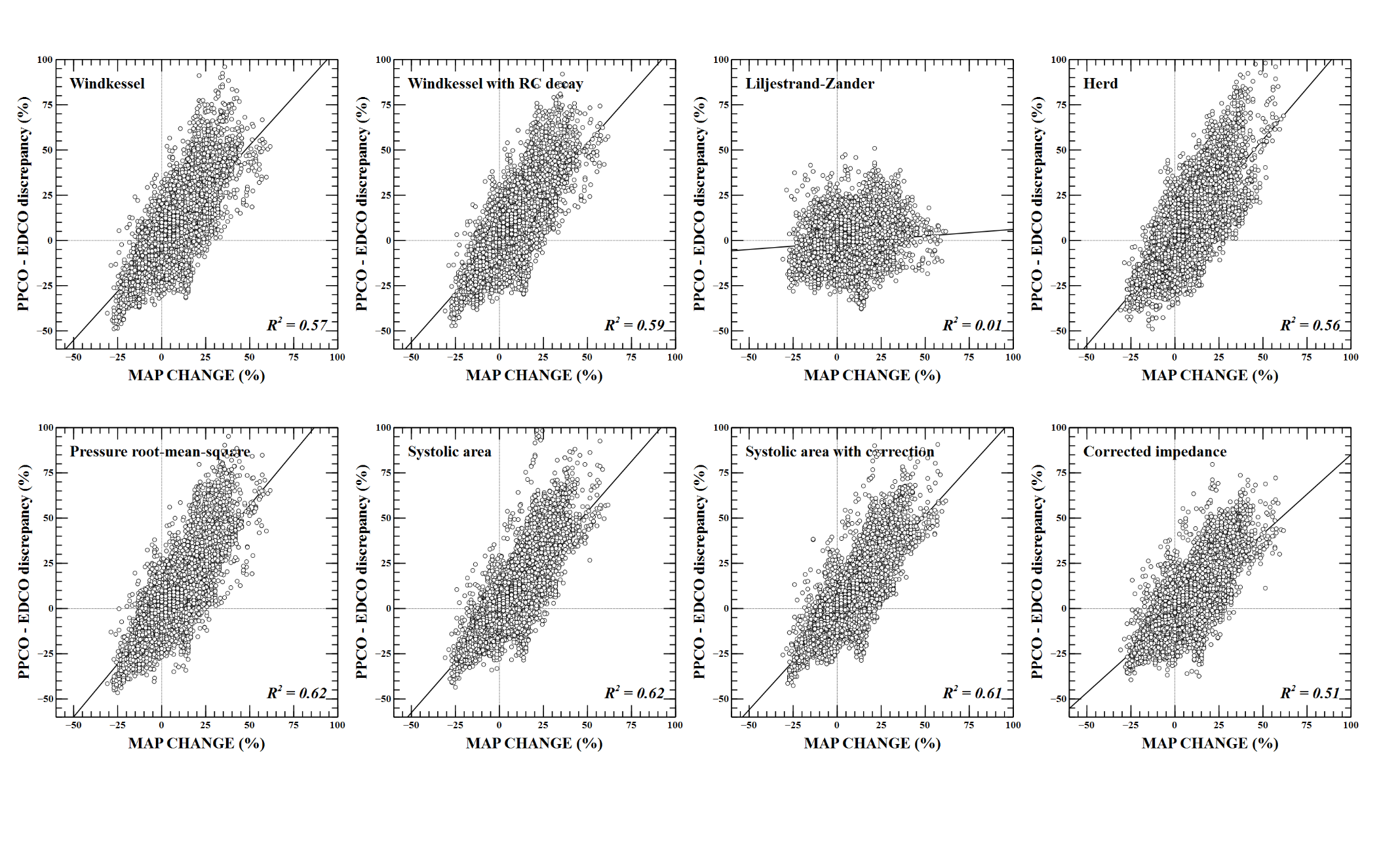** |
| PPCO: pulse pressure-derived cardiac output; EDCO: esophageal Doppler cardiac output; MAP: mean arterial pressure; PPCO-EDCO discrepancy (%): PPCO – EDCO / EDCO. |

| **Figure 4. Influence of total systemic vascular resistance changes on discrepancies between PPCO and EDCO.** |
| --- |
| **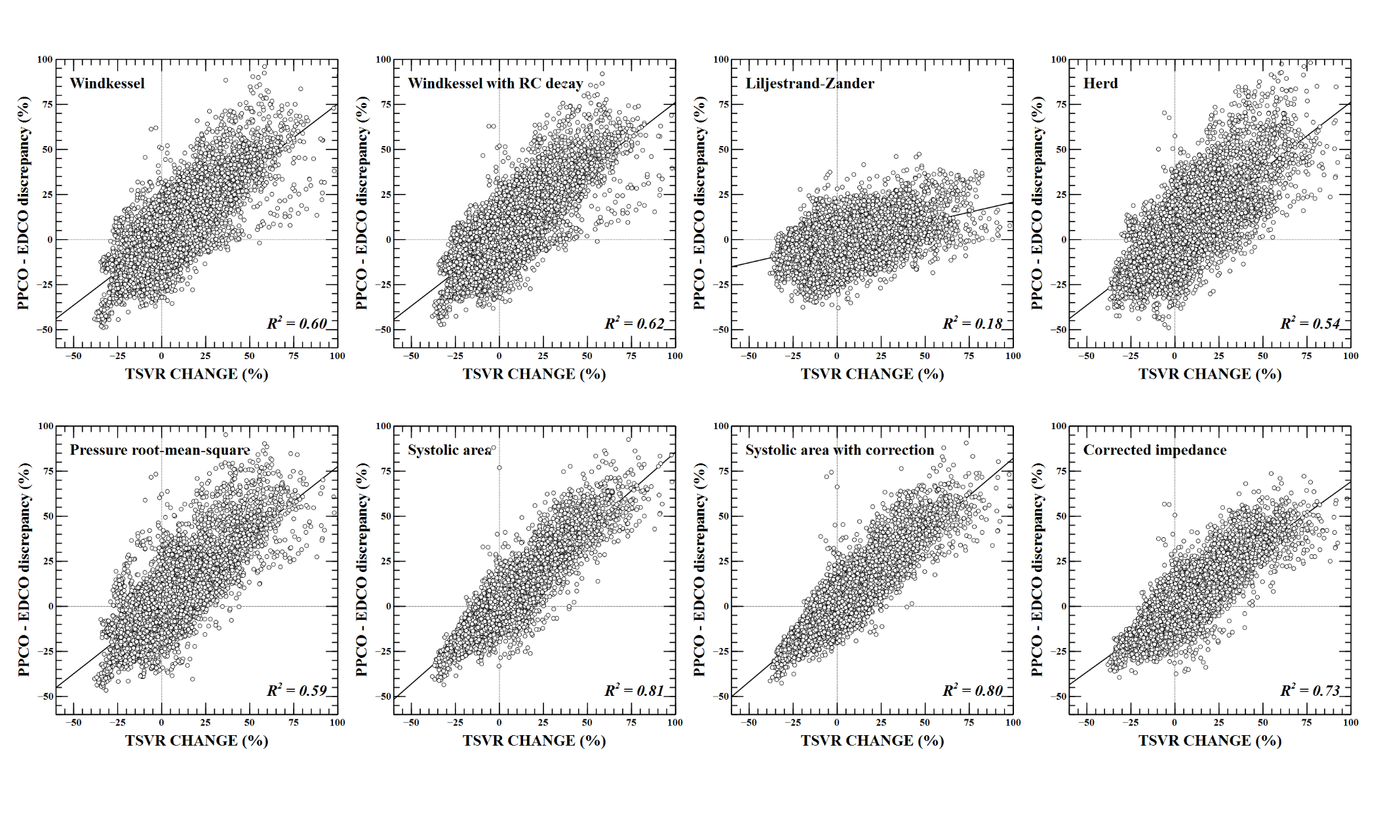** |
| PPCO: pulse pressure-derived cardiac output; EDCO: esophageal Doppler cardiac output; TSVR: total systemic vascular resistance; PPCO-EDCO discrepancy (%): PPCO – EDCO / EDCO. |

| **Figure 5. Influence of net arterial compliance changes on discrepancies between PPCO and EDCO.** |
| --- |
| **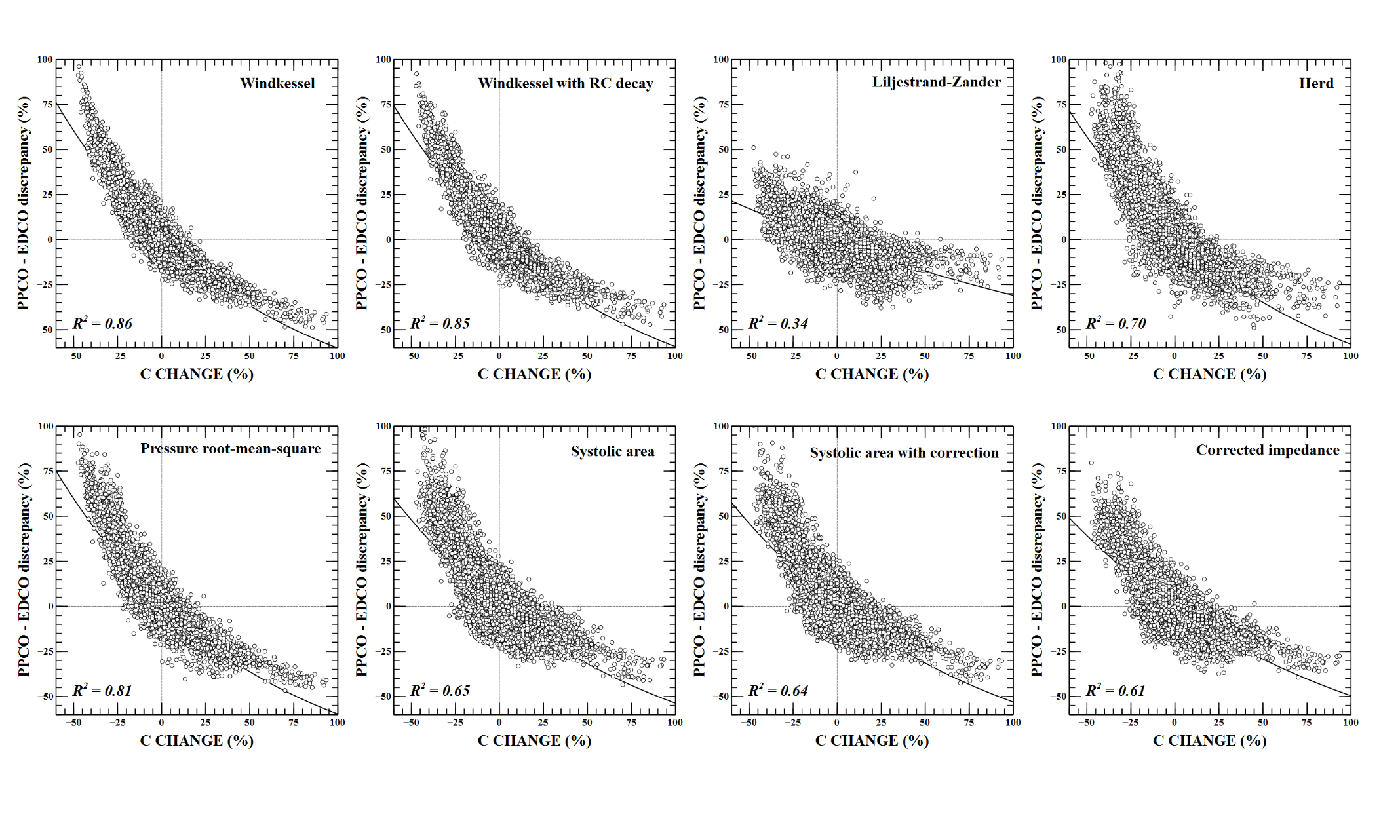** |
| PPCO: pulse pressure-derived cardiac output; EDCO: esophageal Doppler cardiac output; C: net arterial compliance; PPCO-EDCO discrepancy (%): PPCO – EDCO / EDCO. |

| **Figure 6. Influence of effective arterial elastance changes on discrepancies between PPCO and EDCO.** |
| --- |
| **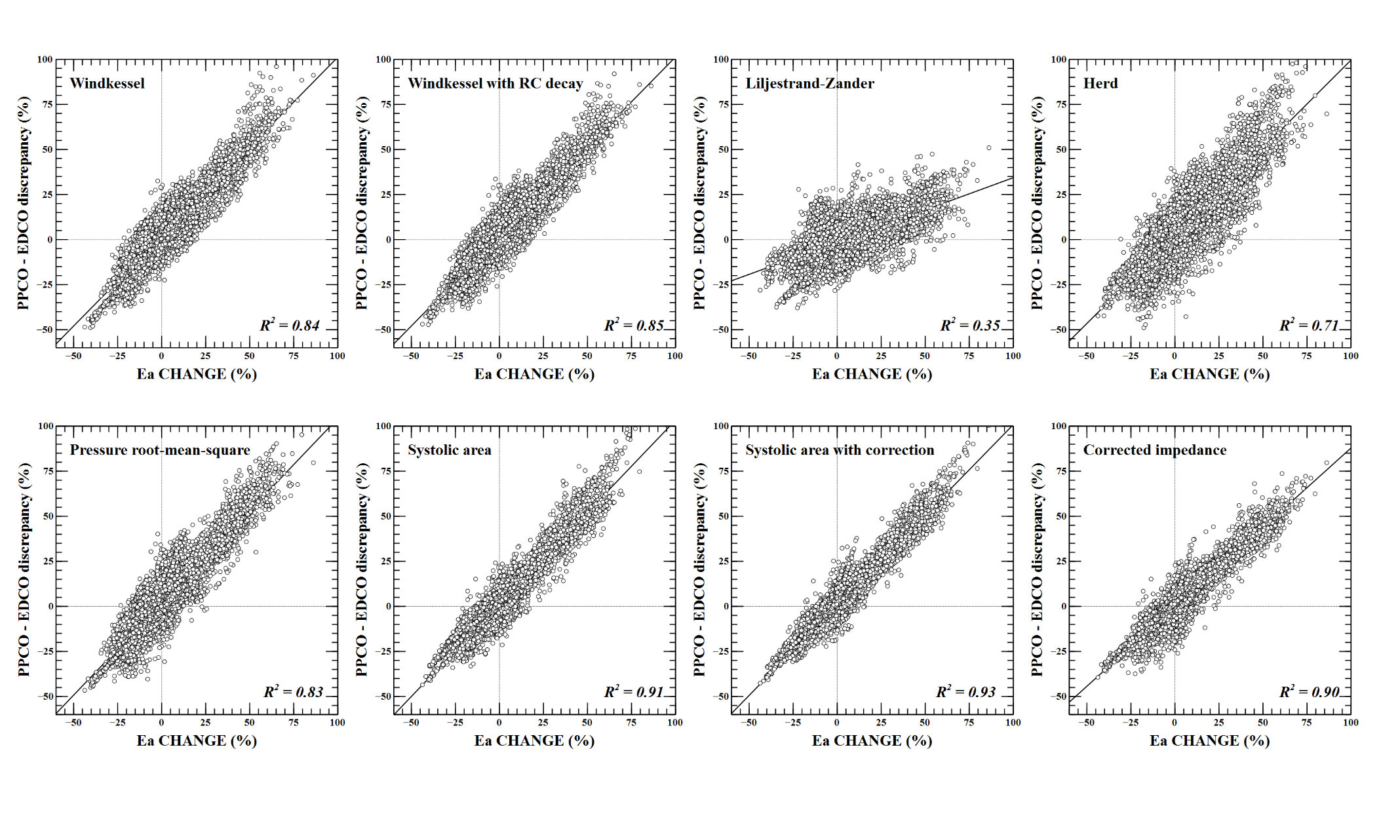** |
| PPCO: pulse pressure-derived cardiac output; EDCO: esophageal Doppler cardiac output; Ea: effective arterial elastance; PPCO-EDCO discrepancy (%): PPCO – EDCO / EDCO. |

| **Figure 7. ROC curves showing the ability of absolute changes on each arterial load parameters to detect an absolute PPCO-EDCO discrepancy ≥ 10% in all studied PPCO algorithms.** | | | |
| --- | --- | --- | --- |
| **Windkessel** | **Windkessel with RC decay** | **Liljestrand-Zander** | **Herd** |
| **0**  **20**  **40**  **60**  **80**  **100**  **0**  **20**  **40**  **60**  **80**  **100**  **100-Specificity**  **Sensitivity**  **C**  **Ea**  **TSVR**  **MAP** | **0**  **20**  **40**  **60**  **80**  **100**  **0**  **20**  **40**  **60**  **80**  **100**  **100-Specificity**  **Sensitivity**  **C**  **Ea**  **TSVR**  **MAP** | **0**  **20**  **40**  **60**  **80**  **100**  **0**  **20**  **40**  **60**  **80**  **100**  **100-Specificity**  **Sensitivity**  **C**  **Ea**  **TSVR**  **MAP** | **0**  **20**  **40**  **60**  **80**  **100**  **0**  **20**  **40**  **60**  **80**  **100**  **100-Specificity**  **Sensitivity**  **C**  **Ea**  **TSVR**  **MAP** |
|  |  |  |  |
| **Pressure root-mean-square** | **Systolic area** | **Systolic area with correction** | **Corrected impedance** |
| **0**  **20**  **40**  **60**  **80**  **100**  **0**  **20**  **40**  **60**  **80**  **100**  **100-Specificity**  **Sensitivity**  **C**  **Ea**  **TSVR**  **MAP** | **0**  **20**  **40**  **60**  **80**  **100**  **0**  **20**  **40**  **60**  **80**  **100**  **100-Specificity**  **Sensitivity**  **C**  **Ea**  **TSVR**  **MAP** | **0**  **20**  **40**  **60**  **80**  **100**  **0**  **20**  **40**  **60**  **80**  **100**  **100-Specificity**  **Sensitivity**  **C**  **Ea**  **TSVR**  **MAP** | **0**  **20**  **40**  **60**  **80**  **100**  **0**  **20**  **40**  **60**  **80**  **100**  **100-Specificity**  **Sensitivity**  **C**  **Ea**  **TSVR**  **MAP** |
| C: net arterial compliance; Ea: effective arterial elastance; MAP: mean arterial pressure; TSVR: total systemic vascular resistance. The values of each ROC curve are shown in table ESM1. Comparison of AUC for each arterial load parameter was significantly different in all algorithms according to De Long et al. method. | | | |

**APPENDIX: Description of all PPCO algorithms tested in the study.**

***Windkessel model***

In its simplest conception, Erlanger *et al.*[[1](#_ENREF_1)] suggested that the arterial system acts as a single elastic chamber in which SV is entirely transferred to generate the arterial pulse pressure (PP; i.e., systolic minus diastolic pressure). Therefore, arterial PP could be used as a surrogate of stroke volume (SV) for estimating cardiac output (CO).

***Windkessel with RC decay***

Burgeoise *et al.*[[2](#_ENREF_2)] proposed this approach to overcome the drainage of blood out into peripheral vascular bed during cardiac ejection (the “systolic run-off”). The actual SV is estimated adding to the measured arterial pressure the pressure leak due to the systolic runoff. This peripheral outflow can be characterized by the arterial pressure decay during diastole as a function of peripheral resistance and assuming a constant compliance. We used a simplified adaptation of the original algorithm, taking the peak of the systolic pressure to the end of the diastole as the components of a mono-exponential decay to determine the draining to peripheral vessels to the total blood flow[[3](#_ENREF_3)].

***Liljestrand and Zander***

Since capacitance of the arterial wall is pressure-dependent and varies in a non-uniform way, arterial pulse pressure should be corrected for a nonlinear arterial compliance. As the pressure increases (and the arterial vessel becomes stiffer), arterial compliance decreases[[4](#_ENREF_4)].

***Herd***

Based upon their empirical results, Herd *et al.* suggested that arterial pulse pressure would be proportional to SV as the cardiac cycle remains constant and that could be calculated as the difference between mean arterial pressure and diastolic blood pressure[[5](#_ENREF_5)].

***Pressure root-mean-square (RMS)***

Unlike other algorithms, the RMS method is not based on the analysis of the morphology of the arterial pressure waveform, but on the principle of conservation of mass and energy. According to the pulse power analysis, SV can be estimated from the nominal SV, computed as the quadratic mean of a standardized volume transformation of arterial pressure waveform. The algorithm used in our study is a very simplified approach to the commercial algorithm of PulseCO method present on the LiDCO device[[6](#_ENREF_6)].

***Systolic area***

Rather than considering the arterial tree as a simple lumped system, arterial circulation can be approached from a distributed model point of view. Based on this assumption, the area under the systolic portion of the arterial waveform (A_sys_) could be considered proportional to SV[[7](#_ENREF_7), [8](#_ENREF_8)].

***Systolic area with correction***

First proposed by Warner *et al.*[[9](#_ENREF_9)] and later revised by Kouchoukos *et al*.[[10](#_ENREF_10)], this method modifies previous algorithm adding a correction factor in order to consider the arterial runoff to the periphery during systole.

***Corrected impedance (cZ)***

The corrected characteristic impedance algorithm (or *cZ* method) suggested by Wesseling *et al.*[[11](#_ENREF_11), [12](#_ENREF_12)] attempts to introduces the concept of aortic input impedance to account the effect of opposition to pulsatile blood flow. This method estimates SV dividing A_sys_ by the aortic input impedance. A correction is also applied in order to account for non-linear pressure dependency of cross sectional area of aorta and for wave reflections from the periphery of the arterial system. In our study, we used the formula from Rauch *et al.*[[13](#_ENREF_13)], which it deviates from the original Wesseling’s algorithm and was actually used on the first version of the PiCCO pulse pressure algorithm[[14](#_ENREF_14)].

**REFERENCES**

1. Erlanger J, Hooker DR: **Experimental study blood-pressure pulse-pressure man**. *Johns Hopkins Hosp Rep* 1904, **12**:145-378.

2. Bourgeois MJ, Gilbert BK, Von Bernuth G, Wood EH: **Continuous determination of beat to beat stroke volume from aortic pressure pulses in the dog**. *Circ Res* 1976, **39**(1):15-24.

3. Sun JX, Reisner AT, Saeed M, Heldt T, Mark RG: **The cardiac output from blood pressure algorithms trial**. *Crit Care Med* 2009, **37**(1):72-80.

4. Liljestrand G, Zander E: **Vergleichende Bestimmungen des Minutenvolumens des Herzens beim Menschen mittels der Stickoxydulmethode und durch Blutdruckmessung**. *Z Ges Exp Med* 1928, **59**:105-122.

5. Herd JA, Leclair NR, Simon W: **Arterial pressure pulse contours during hemorrhage in anesthetized dogs**. *J Appl Physiol* 1966, **21**(6):1864-1868.

6. Sundar S, Panzica P: **LiDCO systems**. *Int Anesthesiol Clin* 2010, **48**(1):87-100.

7. Verdouw PD, Beaune J, Roelandt J, Hugenholtz PG: **Stroke volume from central aortic pressure? A critical assessment of the various formulae as to their clinical value**. *Basic Res Cardiol* 1975, **70**(4):377-389.

8. Jones WB, Hefner LL, Bancroft WH, Jr., Klip W: **Velocity of blood flow and stroke volume obtained from the pressure pulse**. *J Clin Invest* 1959, **38**:2087-2090.

9. Warner HR: **Quantitation of stroke volume changes in man from the central pressure pulse**. *Minn Med* 1954, **37**(2):111-115; passim.

10. Kouchoukos NT, Sheppard LC, McDonald DA: **Estimation of stroke volume in the dog by a pulse contour method**. *Circ Res* 1970, **26**(5):611-623.

11. Wesseling KH, de Wit B, Weber JAP, Ty Smith N: **A simple device for the continuous measurement of cardiac output**. *Adv Cardiovasc Physiol* 1983, **5**(2):16-52.

12. Wesseling KH, Jansen JR, Settels JJ, Schreuder JJ: **Computation of aortic flow from pressure in humans using a nonlinear, three-element model**. *J Appl Physiol* 1993, **74**(5):2566-2573.

13. Rauch H, Muller M, Fleischer F, Bauer H, Martin E, Bottiger BW: **Pulse contour analysis versus thermodilution in cardiac surgery patients**. *Acta Anaesthesiol Scand* 2002, **46**(4):424-429.

14. Godje O, Hoke K, Goetz AE, Felbinger TW, Reuter DA, Reichart B, Friedl R, Hannekum A, Pfeiffer UJ: **Reliability of a new algorithm for continuous cardiac output determination by pulse-contour analysis during hemodynamic instability**. *Crit Care Med* 2002, **30**(1):52-58.
